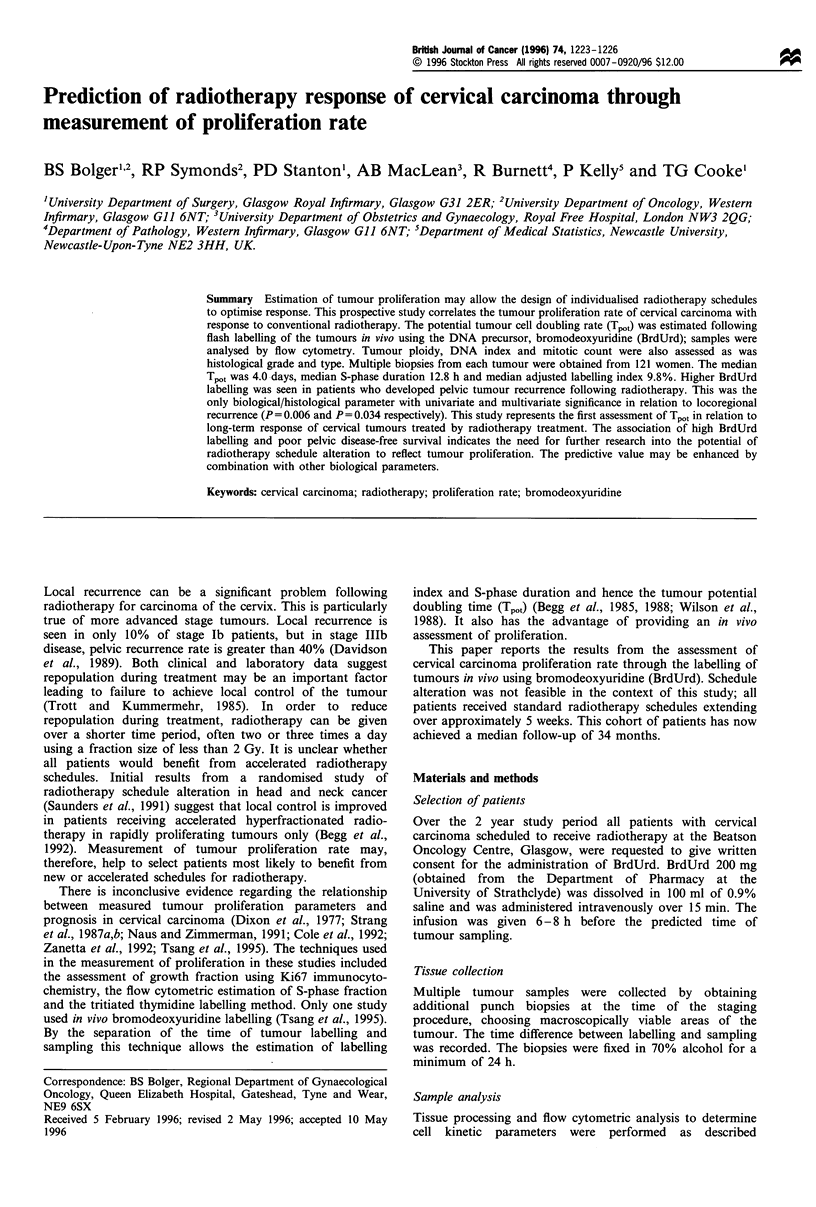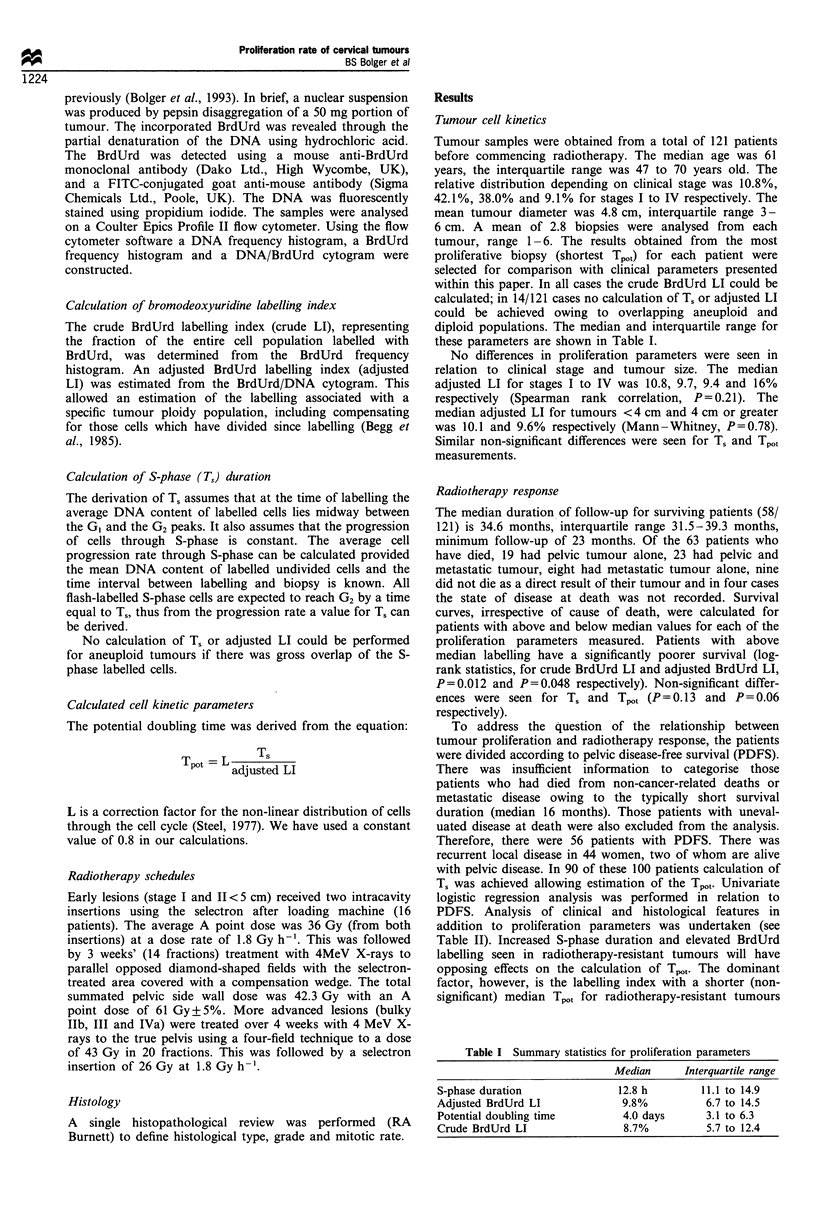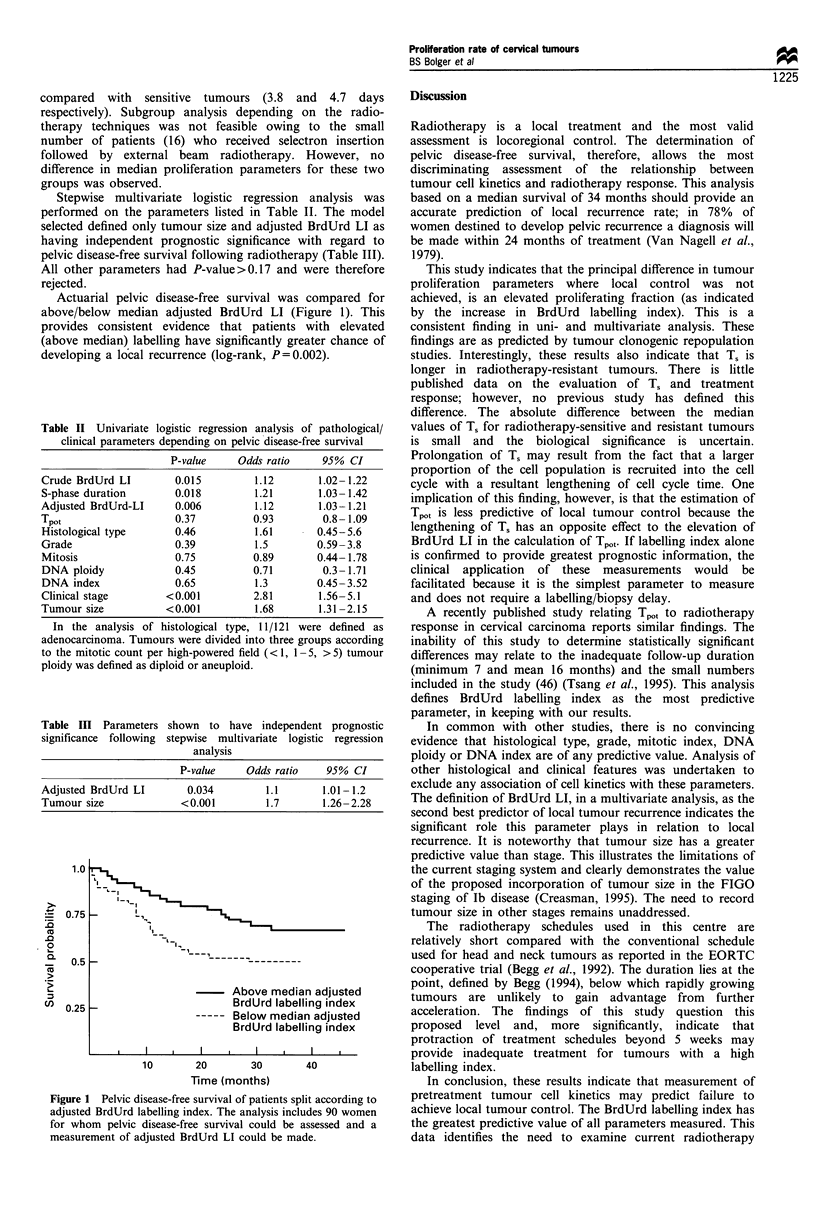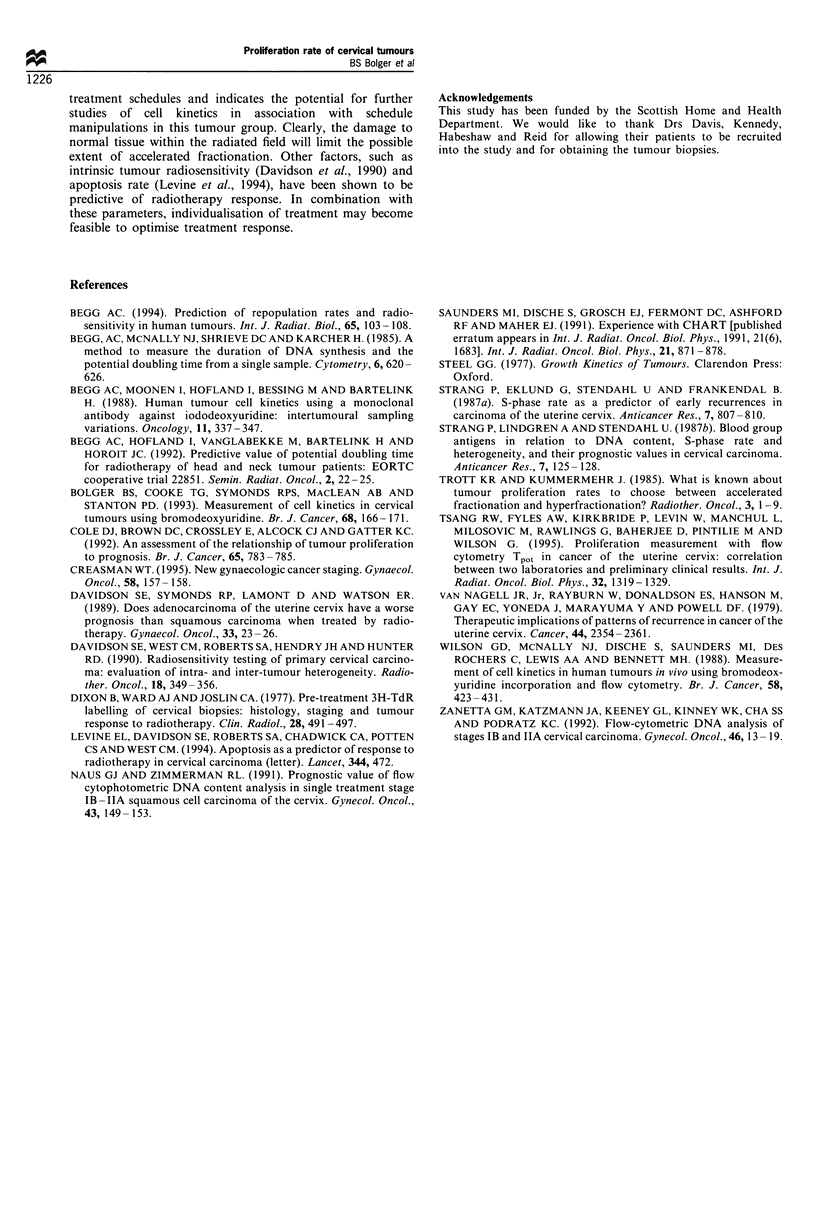# Prediction of radiotherapy response of cervical carcinoma through measurement of proliferation rate.

**DOI:** 10.1038/bjc.1996.520

**Published:** 1996-10

**Authors:** B. S. Bolger, R. P. Symonds, P. D. Stanton, A. B. MacLean, R. Burnett, P. Kelly, T. G. Cooke

**Affiliations:** University Department of Surgery, Glasgow Royal Infirmary, UK.

## Abstract

Estimation of tumour proliferation may allow the design of individualised radiotherapy schedules to optimise response. This prospective study correlates the tumour proliferation rate of cervical carcinoma with response to conventional radiotherapy. The potential tumour cell doubling rate (Tpot) was estimated following flash labelling of the tumours in vivo using the DNA precursor, bromodeoxyuridine (BrdUrd); samples were analysed by flow cytometry. Tumour ploidy, DNA index and mitotic count were also assessed as was histological grade and type. Multiple biopsies from each tumour were obtained from 121 women. The median Tpot was 4.0 days, median S-phase duration 12.8 h and median adjusted labelling index 9.8%. Higher BrdUrd labelling was seen in patients who developed pelvic tumour recurrence following radiotherapy. This was the only biological/histological parameter with univariate and multivariate significance in relation to locoregional recurrence (P = 0.006 and P = 0.034 respectively). This study represents the first assessment of Tpot in relation to long-term response of cervical tumours treated by radiotherapy treatment. The association of high BrdUrd labelling and poor pelvic disease-free survival indicates the need for further research into the potential of radiotherapy schedule alteration to reflect tumour proliferation. The predictive value may be enhanced by combination with other biological parameters.


British Journal of Cancer (1996) 74, 1223-1226
© 1996 Stockton Press All rights reserved 0007-0920/96 $12.00

### Prediction of radiotherapy response of cervical carcinoma through measurement of proliferation rate

BS Bolger[1,2], RP Symonds[2], PD Stanton[1], AB MacLean[3], R Burnett[4], P Kelly[5] and TG Cooke[1]

[1]*University Department of Surgery, Glasgow Royal Infirmary, Glasgow G31 2ER;* [2]*University Department of Oncology, Western Infirmary, Glasgow G11 6NT;* [3]*University Department of Obstetrics and Gynaecology, Royal Free Hospital, London NW3 2QG;* [4]*Department of Pathology, Western Infirmary, Glasgow G11 6NT;* [5]*Department of Medical Statistics, Newcastle University, Newcastle-Upon-Tyne NE2 3HH, UK.*

**Summary** Estimation of tumour proliferation may allow the design of individualised radiotherapy schedules to optimise response. This prospective study correlates the tumour proliferation rate of cervical carcinoma with response to conventional radiotherapy. The potential tumour cell doubling rate ($T_{pot}$) was estimated following flash labelling of the tumours *in vivo* using the DNA precursor, bromodeoxyuridine (BrdUrd); samples were analysed by flow cytometry. Tumour ploidy, DNA index and mitotic count were also assessed as was histological grade and type. Multiple biopsies from each tumour were obtained from 121 women. The median $T_{pot}$ was 4.0 days, median S-phase duration 12.8 h and median adjusted labelling index 9.8%. Higher BrdUrd labelling was seen in patients who developed pelvic tumour recurrence following radiotherapy. This was the only biological/histological parameter with univariate and multivariate significance in relation to locoregional recurrence ($P=0.006$ and $P=0.034$ respectively). This study represents the first assessment of $T_{pot}$ in relation to long-term response of cervical tumours treated by radiotherapy treatment. The association of high BrdUrd labelling and poor pelvic disease-free survival indicates the need for further research into the potential of radiotherapy schedule alteration to reflect tumour proliferation. The predictive value may be enhanced by combination with other biological parameters.

**Keywords:** cervical carcinoma; radiotherapy; proliferation rate; bromodeoxyuridine

Local recurrence can be a significant problem following radiotherapy for carcinoma of the cervix. This is particularly true of more advanced stage tumours. Local recurrence is seen in only 10% of stage Ib patients, but in stage IIIb disease, pelvic recurrence rate is greater than 40% (Davidson *et al.*, 1989). Both clinical and laboratory data suggest repopulation during treatment may be an important factor leading to failure to achieve local control of the tumour (Trott and Kummermehr, 1985). In order to reduce repopulation during treatment, radiotherapy can be given over a shorter time period, often two or three times a day using a fraction size of less than 2 Gy. It is unclear whether all patients would benefit from accelerated radiotherapy schedules. Initial results from a randomised study of radiotherapy schedule alteration in head and neck cancer (Saunders *et al.*, 1991) suggest that local control is improved in patients receiving accelerated hyperfractionated radiotherapy in rapidly proliferating tumours only (Begg *et al.*, 1992). Measurement of tumour proliferation rate may, therefore, help to select patients most likely to benefit from new or accelerated schedules for radiotherapy.

There is inconclusive evidence regarding the relationship between measured tumour proliferation parameters and prognosis in cervical carcinoma (Dixon *et al.*, 1977; Strang *et al.*, 1987*a,b*; Naus and Zimmerman, 1991; Cole *et al.*, 1992; Zanetta *et al.*, 1992; Tsang *et al.*, 1995). The techniques used in the measurement of proliferation in these studies included the assessment of growth fraction using Ki67 immunocytochemistry, the flow cytometric estimation of S-phase fraction and the tritiated thymidine labelling method. Only one study used *in vivo* bromodeoxyuridine labelling (Tsang *et al.*, 1995). By the separation of the time of tumour labelling and sampling this technique allows the estimation of labelling index and S-phase duration and hence the tumour potential doubling time ($T_{pot}$) (Begg *et al.*, 1985, 1988; Wilson *et al.*, 1988). It also has the advantage of providing an *in vivo* assessment of proliferation.

This paper reports the results from the assessment of cervical carcinoma proliferation rate through the labelling of tumours *in vivo* using bromodeoxyuridine (BrdUrd). Schedule alteration was not feasible in the context of this study; all patients received standard radiotherapy schedules extending over approximately 5 weeks. This cohort of patients has now achieved a median follow-up of 34 months.

### Materials and methods

### *Selection of patients*

Over the 2 year study period all patients with cervical carcinoma scheduled to receive radiotherapy at the Beatson Oncology Centre, Glasgow, were requested to give written consent for the administration of BrdUrd. BrdUrd 200 mg (obtained from the Department of Pharmacy at the University of Strathclyde) was dissolved in 100 ml of 0.9% saline and was administered intravenously over 15 min. The infusion was given 6–8 h before the predicted time of tumour sampling.

### *Tissue collection*

Multiple tumour samples were collected by obtaining additional punch biopsies at the time of the staging procedure, choosing macroscopically viable areas of the tumour. The time difference between labelling and sampling was recorded. The biopsies were fixed in 70% alcohol for a minimum of 24 h.

### *Sample analysis*

Tissue processing and flow cytometric analysis to determine cell kinetic parameters were performed as described

Correspondence: BS Bolger, Regional Department of Gynaecological Oncology, Queen Elizabeth Hospital, Gateshead, Tyne and Wear, NE9 6SX
Received 5 February 1996; revised 2 May 1996; accepted 10 May 1996

previously (Bolger et al., 1993). In brief, a nuclear suspension was produced by pepsin disaggregation of a 50 mg portion of tumour. The incorporated BrdUrd was revealed through the partial denaturation of the DNA using hydrochloric acid. The BrdUrd was detected using a mouse anti-BrdUrd monoclonal antibody (Dako Ltd., High Wycombe, UK), and a FITC-conjugated goat anti-mouse antibody (Sigma Chemicals Ltd., Poole, UK). The DNA was fluorescently stained using propidium iodide. The samples were analysed on a Coulter Epics Profile II flow cytometer. Using the flow cytometer software a DNA frequency histogram, a BrdUrd frequency histogram and a DNA/BrdUrd cytogram were constructed.

### *Calculation of bromodeoxyuridine labelling index*

The crude BrdUrd labelling index (crude LI), representing the fraction of the entire cell population labelled with BrdUrd, was determined from the BrdUrd frequency histogram. An adjusted BrdUrd labelling index (adjusted LI) was estimated from the BrdUrd/DNA cytogram. This allowed an estimation of the labelling associated with a specific tumour ploidy population, including compensating for those cells which have divided since labelling (Begg et al., 1985).

### *Calculation of S-phase ($T_s$) duration*

The derivation of $T_s$ assumes that at the time of labelling the average DNA content of labelled cells lies midway between the $G_1$ and the $G_2$ peaks. It also assumes that the progression of cells through S-phase is constant. The average cell progression rate through S-phase can be calculated provided the mean DNA content of labelled undivided cells and the time interval between labelling and biopsy is known. All flash-labelled S-phase cells are expected to reach $G_2$ by a time equal to $T_s$, thus from the progression rate a value for $T_s$ can be derived.

No calculation of $T_s$ or adjusted LI could be performed for aneuploid tumours if there was gross overlap of the S-phase labelled cells.

### *Calculated cell kinetic parameters*

The potential doubling time was derived from the equation:

$$T_{pot} = L\frac{T_s}{\text{adjusted LI}}$$

L is a correction factor for the non-linear distribution of cells through the cell cycle (Steel, 1977). We have used a constant value of 0.8 in our calculations.

### *Radiotherapy schedules*

Early lesions (stage I and II < 5 cm) received two intracavity insertions using the selectron after loading machine (16 patients). The average A point dose was 36 Gy (from both insertions) at a dose rate of 1.8 Gy $h^{-1}$. This was followed by 3 weeks' (14 fractions) treatment with 4MeV X-rays to parallel opposed diamond-shaped fields with the selectron-treated area covered with a compensation wedge. The total summated pelvic side wall dose was 42.3 Gy with an A point dose of 61 Gy $\pm$ 5%. More advanced lesions (bulky IIb, III and IVa) were treated over 4 weeks with 4 MeV X-rays to the true pelvis using a four-field technique to a dose of 43 Gy in 20 fractions. This was followed by a selectron insertion of 26 Gy at 1.8 Gy $h^{-1}$.

### *Histology*

A single histopathological review was performed (RA Burnett) to define histological type, grade and mitotic rate.

### Results

### *Tumour cell kinetics*

Tumour samples were obtained from a total of 121 patients before commencing radiotherapy. The median age was 61 years, the interquartile range was 47 to 70 years old. The relative distribution depending on clinical stage was 10.8%, 42.1%, 38.0% and 9.1% for stages I to IV respectively. The mean tumour diameter was 4.8 cm, interquartile range 3–6 cm. A mean of 2.8 biopsies were analysed from each tumour, range 1–6. The results obtained from the most proliferative biopsy (shortest $T_{pot}$) for each patient were selected for comparison with clinical parameters presented within this paper. In all cases the crude BrdUrd LI could be calculated; in 14/121 cases no calculation of $T_s$ or adjusted LI could be achieved owing to overlapping aneuploid and diploid populations. The median and interquartile range for these parameters are shown in Table I.

No differences in proliferation parameters were seen in relation to clinical stage and tumour size. The median adjusted LI for stages I to IV was 10.8, 9.7, 9.4 and 16% respectively (Spearman rank correlation, $P = 0.21$). The median adjusted LI for tumours < 4 cm and 4 cm or greater was 10.1 and 9.6% respectively (Mann–Whitney, $P = 0.78$). Similar non-significant differences were seen for $T_s$ and $T_{pot}$ measurements.

### *Radiotherapy response*

The median duration of follow-up for surviving patients (58/121) is 34.6 months, interquartile range 31.5–39.3 months, minimum follow-up of 23 months. Of the 63 patients who have died, 19 had pelvic tumour alone, 23 had pelvic and metastatic tumour, eight had metastatic tumour alone, nine did not die as a direct result of their tumour and in four cases the state of disease at death was not recorded. Survival curves, irrespective of cause of death, were calculated for patients with above and below median values for each of the proliferation parameters measured. Patients with above median labelling have a significantly poorer survival (log-rank statistics, for crude BrdUrd LI and adjusted BrdUrd LI, $P = 0.012$ and $P = 0.048$ respectively). Non-significant differences were seen for $T_s$ and $T_{pot}$ ($P = 0.13$ and $P = 0.06$ respectively).

To address the question of the relationship between tumour proliferation and radiotherapy response, the patients were divided according to pelvic disease-free survival (PDFS). There was insufficient information to categorise those patients who had died from non-cancer-related deaths or metastatic disease owing to the typically short survival duration (median 16 months). Those patients with unevaluated disease at death were also excluded from the analysis. Therefore, there were 56 patients with PDFS. There was recurrent local disease in 44 women, two of whom are alive with pelvic disease. In 90 of these 100 patients calculation of $T_s$ was achieved allowing estimation of the $T_{pot}$. Univariate logistic regression analysis was performed in relation to PDFS. Analysis of clinical and histological features in addition to proliferation parameters was undertaken (see Table II). Increased S-phase duration and elevated BrdUrd labelling seen in radiotherapy-resistant tumours will have opposing effects on the calculation of $T_{pot}$. The dominant factor, however, is the labelling index with a shorter (non-significant) median $T_{pot}$ for radiotherapy-resistant tumours

**Table I** Summary statistics for proliferation parameters

| | *Median* | *Interquartile range* |
|---|---|---|
| S-phase duration | 12.8 h | 11.1 to 14.9 |
| Adjusted BrdUrd LI | 9.8% | 6.7 to 14.5 |
| Potential doubling time | 4.0 days | 3.1 to 6.3 |
| Crude BrdUrd LI | 8.7% | 5.7 to 12.4 |

compared with sensitive tumours (3.8 and 4.7 days respectively). Subgroup analysis depending on the radiotherapy techniques was not feasible owing to the small number of patients (16) who received selectron insertion followed by external beam radiotherapy. However, no difference in median proliferation parameters for these two groups was observed.

Stepwise multivariate logistic regression analysis was performed on the parameters listed in Table II. The model selected defined only tumour size and adjusted BrdUrd LI as having independent prognostic significance with regard to pelvic disease-free survival following radiotherapy (Table III). All other parameters had *P*-value>0.17 and were therefore rejected.

Actuarial pelvic disease-free survival was compared for above/below median adjusted BrdUrd LI (Figure 1). This provides consistent evidence that patients with elevated (above median) labelling have significantly greater chance of developing a local recurrence (log-rank, $P=0.002$).

**Table II** Univariate logistic regression analysis of pathological/clinical parameters depending on pelvic disease-free survival

| | P-*value* | *Odds ratio* | *95% CI* |
|---|---|---|---|
| Crude BrdUrd LI | 0.015 | 1.12 | 1.02–1.22 |
| S-phase duration | 0.018 | 1.21 | 1.03–1.42 |
| Adjusted BrdUrd-LI | 0.006 | 1.12 | 1.03–1.21 |
| $T_{pot}$ | 0.37 | 0.93 | 0.8–1.09 |
| Histological type | 0.46 | 1.61 | 0.45–5.6 |
| Grade | 0.39 | 1.5 | 0.59–3.8 |
| Mitosis | 0.75 | 0.89 | 0.44–1.78 |
| DNA ploidy | 0.45 | 0.71 | 0.3–1.71 |
| DNA index | 0.65 | 1.3 | 0.45–3.52 |
| Clinical stage | <0.001 | 2.81 | 1.56–5.1 |
| Tumour size | <0.001 | 1.68 | 1.31–2.15 |

In the analysis of histological type, 11/121 were defined as adenocarcinoma. Tumours were divided into three groups according to the mitotic count per high-powered field (<1, 1–5, >5) tumour ploidy was defined as diploid or aneuploid.

**Table III** Parameters shown to have independent prognostic significance following stepwise multivariate logistic regression analysis

| | P-*value* | *Odds ratio* | *95% CI* |
|---|---|---|---|
| Adjusted BrdUrd LI | 0.034 | 1.1 | 1.01–1.2 |
| Tumour size | <0.001 | 1.7 | 1.26–2.28 |

**Figure 1** Pelvic disease-free survival of patients split according to adjusted BrdUrd labelling index. The analysis includes 90 women for whom pelvic disease-free survival could be assessed and a measurement of adjusted BrdUrd LI could be made.

### Discussion

Radiotherapy is a local treatment and the most valid assessment is locoregional control. The determination of pelvic disease-free survival, therefore, allows the most discriminating assessment of the relationship between tumour cell kinetics and radiotherapy response. This analysis based on a median survival of 34 months should provide an accurate prediction of local recurrence rate; in 78% of women destined to develop pelvic recurrence a diagnosis will be made within 24 months of treatment (Van Nagell *et al.*, 1979).

This study indicates that the principal difference in tumour proliferation parameters where local control was not achieved, is an elevated proliferating fraction (as indicated by the increase in BrdUrd labelling index). This is a consistent finding in uni- and multivariate analysis. These findings are as predicted by tumour clonogenic repopulation studies. Interestingly, these results also indicate that $T_s$ is longer in radiotherapy-resistant tumours. There is little published data on the evaluation of $T_s$ and treatment response; however, no previous study has defined this difference. The absolute difference between the median values of $T_s$ for radiotherapy-sensitive and resistant tumours is small and the biological significance is uncertain. Prolongation of $T_s$ may result from the fact that a larger proportion of the cell population is recruited into the cell cycle with a resultant lengthening of cell cycle time. One implication of this finding, however, is that the estimation of $T_{pot}$ is less predictive of local tumour control because the lengthening of $T_s$ has an opposite effect to the elevation of BrdUrd LI in the calculation of $T_{pot}$. If labelling index alone is confirmed to provide greatest prognostic information, the clinical application of these measurements would be facilitated because it is the simplest parameter to measure and does not require a labelling/biopsy delay.

A recently published study relating $T_{pot}$ to radiotherapy response in cervical carcinoma reports similar findings. The inability of this study to determine statistically significant differences may relate to the inadequate follow-up duration (minimum 7 and mean 16 months) and the small numbers included in the study (46) (Tsang *et al.*, 1995). This analysis defines BrdUrd labelling index as the most predictive parameter, in keeping with our results.

In common with other studies, there is no convincing evidence that histological type, grade, mitotic index, DNA ploidy or DNA index are of any predictive value. Analysis of other histological and clinical features was undertaken to exclude any association of cell kinetics with these parameters. The definition of BrdUrd LI, in a multivariate analysis, as the second best predictor of local tumour recurrence indicates the significant role this parameter plays in relation to local recurrence. It is noteworthy that tumour size has a greater predictive value than stage. This illustrates the limitations of the current staging system and clearly demonstrates the value of the proposed incorporation of tumour size in the FIGO staging of Ib disease (Creasman, 1995). The need to record tumour size in other stages remains unaddressed.

The radiotherapy schedules used in this centre are relatively short compared with the conventional schedule used for head and neck tumours as reported in the EORTC cooperative trial (Begg *et al.*, 1992). The duration lies at the point, defined by Begg (1994), below which rapidly growing tumours are unlikely to gain advantage from further acceleration. The findings of this study question this proposed level and, more significantly, indicate that protraction of treatment schedules beyond 5 weeks may provide inadequate treatment for tumours with a high labelling index.

In conclusion, these results indicate that measurement of pretreatment tumour cell kinetics may predict failure to achieve local tumour control. The BrdUrd labelling index has the greatest predictive value of all parameters measured. This data identifies the need to examine current radiotherapy treatment schedules and indicates the potential for further studies of cell kinetics in association with schedule manipulations in this tumour group. Clearly, the damage to normal tissue within the radiated field will limit the possible extent of accelerated fractionation. Other factors, such as intrinsic tumour radiosensitivity (Davidson *et al.*, 1990) and apoptosis rate (Levine *et al.*, 1994), have been shown to be predictive of radiotherapy response. In combination with these parameters, individualisation of treatment may become feasible to optimise treatment response.

**Acknowledgements**
This study has been funded by the Scottish Home and Health Department. We would like to thank Drs Davis, Kennedy, Habeshaw and Reid for allowing their patients to be recruited into the study and for obtaining the tumour biopsies.

### References

BEGG AC. (1994). Prediction of repopulation rates and radiosensitivity in human tumours. *Int. J. Radiat. Biol.*, **65,** 103–108.

BEGG, AC, MCNALLY NJ, SHRIEVE DC AND KARCHER H. (1985). A method to measure the duration of DNA synthesis and the potential doubling time from a single sample. *Cytometry*, **6,** 620–626.

BEGG AC, MOONEN I, HOFLAND I, BESSING M AND BARTELINK H. (1988). Human tumour cell kinetics using a monoclonal antibody against iododeoxyuridine: intertumoural sampling variations. *Oncology*, **11,** 337–347.

BEGG AC, HOFLAND I, VANGLABEKKE M, BARTELINK H AND HOROIT JC. (1992). Predictive value of potential doubling time for radiotherapy of head and neck tumour patients: EORTC cooperative trial 22851. *Semin. Radiat. Oncol.*, **2,** 22–25.

BOLGER BS, COOKE TG, SYMONDS RPS, MACLEAN AB AND STANTON PD. (1993). Measurement of cell kinetics in cervical tumours using bromodeoxyuridine. *Br. J. Cancer*, **68,** 166–171.

COLE DJ, BROWN DC, CROSSLEY E, ALCOCK CJ AND GATTER KC. (1992). An assessment of the relationship of tumour proliferation to prognosis. *Br. J. Cancer*, **65,** 783–785.

CREASMAN WT. (1995). New gynaecologic cancer staging. *Gynaecol. Oncol.*, **58,** 157–158.

DAVIDSON SE, SYMONDS RP, LAMONT D AND WATSON ER. (1989). Does adenocarcinoma of the uterine cervix have a worse prognosis than squamous carcinoma when treated by radiotherapy. *Gynaecol. Oncol.*, **33,** 23–26.

DAVIDSON SE, WEST CM, ROBERTS SA, HENDRY JH AND HUNTER RD. (1990). Radiosensitivity testing of primary cervical carcinoma: evaluation of intra- and inter-tumour heterogeneity. *Radiother. Oncol.*, **18,** 349–356.

DIXON B, WARD AJ AND JOSLIN CA. (1977). Pre-treatment 3H-TdR labelling of cervical biopsies: histology, staging and tumour response to radiotherapy. *Clin. Radiol.*, **28,** 491–497.

LEVINE EL, DAVIDSON SE, ROBERTS SA, CHADWICK CA, POTTEN CS AND WEST CM. (1994). Apoptosis as a predictor of response to radiotherapy in cervical carcinoma (letter). *Lancet*, **344,** 472.

NAUS GJ AND ZIMMERMAN RL. (1991). Prognostic value of flow cytophotometric DNA content analysis in single treatment stage IB–IIA squamous cell carcinoma of the cervix. *Gynecol. Oncol.*, **43,** 149–153.

SAUNDERS MI, DISCHE S, GROSCH EJ, FERMONT DC, ASHFORD RF AND MAHER EJ. (1991). Experience with CHART [published erratum appears in *Int. J. Radiat. Oncol. Biol. Phys.*, 1991, 21(6), 1683]. *Int. J. Radiat. Oncol. Biol. Phys.*, **21,** 871–878.

STEEL GG. (1977). *Growth Kinetics of Tumours.* Clarendon Press: Oxford.

STRANG P, EKLUND G, STENDAHL U AND FRANKENDAL B. (1987*a*). S-phase rate as a predictor of early recurrences in carcinoma of the uterine cervix. *Anticancer Res.*, **7,** 807–810.

STRANG P, LINDGREN A AND STENDAHL U. (1987*b*). Blood group antigens in relation to DNA content, S-phase rate and heterogeneity, and their prognostic values in cervical carcinoma. *Anticancer Res.*, **7,** 125–128.

TROTT KR AND KUMMERMEHR J. (1985). What is known about tumour proliferation rates to choose between accelerated fractionation and hyperfractionation? *Radiother. Oncol.*, **3,** 1–9.

TSANG RW, FYLES AW, KIRKBRIDE P, LEVIN W, MANCHUL L, MILOSOVIC M, RAWLINGS G, BAHERJEE D, PINTILIE M AND WILSON G. (1995). Proliferation measurement with flow cytometry $T_{pot}$ in cancer of the uterine cervix: correlation between two laboratories and preliminary clinical results. *Int. J. Radiat. Oncol. Biol. Phys.*, **32,** 1319–1329.

VAN NAGELL JR, JR, RAYBURN W, DONALDSON ES, HANSON M, GAY EC, YONEDA J, MARAYUMA Y AND POWELL DF. (1979). Therapeutic implications of patterns of recurrence in cancer of the uterine cervix. *Cancer*, **44,** 2354–2361.

WILSON GD, MCNALLY NJ, DISCHE S, SAUNDERS MI, DES ROCHERS C, LEWIS AA AND BENNETT MH. (1988). Measurement of cell kinetics in human tumours *in vivo* using bromodeoxyuridine incorporation and flow cytometry. *Br. J. Cancer*, **58,** 423–431.

ZANETTA GM, KATZMANN JA, KEENEY GL, KINNEY WK, CHA SS AND PODRATZ KC. (1992). Flow-cytometric DNA analysis of stages IB and IIA cervical carcinoma. *Gynecol. Oncol.*, **46,** 13–19.